# Machine learning decision tree models for multiclass classification of common malignant brain tumors using perfusion and spectroscopy MRI data

**DOI:** 10.3389/fonc.2023.1089998

**Published:** 2023-08-08

**Authors:** Rodolphe Vallée, Jean-Noël Vallée, Carole Guillevin, Athéna Lallouette, Clément Thomas, Guillaume Rittano, Michel Wager, Rémy Guillevin, Alexandre Vallée

**Affiliations:** ^1^ Interdisciplinary Laboratory in Neurosciences, Physiology and Psychology (LINP2), Université Paris Lumière (UPL), Paris Nanterre University, Nanterre, France; ^2^ Laboratory of Mathematics and Applications (LMA) Centre National de la Recherche Scientifique - Unité Mixte de Recherche (CNRS UMR)7348, i3M-DACTIM-MIH (Data Analysis and Computations Through Imaging Modeling - Mathematics, Image, Health), Poitiers University, Poitiers, France; ^3^ Glaucoma Research Center, Swiss Visio Network, Lausanne, Switzerland; ^4^ Diagnostic and Functional Neuroradiology and Brain stimulation Department, 15-20 National Vision Hospital of Paris - Paris University Hospital Center, University of PARIS-SACLAY - UVSQ, Paris, France; ^5^ Radiology Department, Poitiers University Hospital, Poitiers University, Poitiers, France; ^6^ Center of Genève Ophtalmologie, Geneve, Switzerland; ^7^ Radiology Department, Hopital Riveira Chablais, Rennaz, Switzerland; ^8^ Neurosurgery Department, Poitiers University Hospital, Poitiers University, Poitiers, France; ^9^ Department of Epidemiology and Public Health, Foch Hospital, Suresnes, France

**Keywords:** classification and regression tree (CART), multiclass classification, lymphoma, glioblastoma, metastasis

## Abstract

**Background:**

To investigate the contribution of machine learning decision tree models applied to perfusion and spectroscopy MRI for multiclass classification of lymphomas, glioblastomas, and metastases, and then to bring out the underlying key pathophysiological processes involved in the hierarchization of the decision-making algorithms of the models

**Methods:**

From 2013 to 2020, 180 consecutive patients with histopathologically proved lymphomas (n = 77), glioblastomas (n = 45), and metastases (n = 58) were included in machine learning analysis after undergoing MRI. The perfusion parameters (rCBV_max_, PSR_max_) and spectroscopic concentration ratios (lac/Cr, Cho/NAA, Cho/Cr, and lip/Cr) were applied to construct Classification and Regression Tree (CART) models for multiclass classification of these brain tumors. A 5-fold random cross validation was performed on the dataset.

**Results:**

The decision tree model thus constructed successfully classified all 3 tumor types with a performance (AUC) of 0.98 for PCNSLs, 0.98 for GBM and 1.00 for METs. The model accuracy was 0.96 with a RSquare of 0.887. Five rules of classifier combinations were extracted with a predicted probability from 0.907 to 0.989 for that end nodes of the decision tree for tumor multiclass classification. In hierarchical order of importance, the root node (Cho/NAA) in the decision tree algorithm was primarily based on the proliferative, infiltrative, and neuronal destructive characteristics of the tumor, the internal node (PSRmax), on tumor tissue capillary permeability characteristics, and the end node (Lac/Cr or Cho/Cr), on tumor energy glycolytic (Warburg effect), or on membrane lipid tumor metabolism.

**Conclusion:**

Our study shows potential implementation of machine learning decision tree model algorithms based on a hierarchical, convenient, and personalized use of perfusion and spectroscopy MRI data for multiclass classification of these brain tumors.

## Introduction

Glioblastomas (GBMs), primary central nervous system lymphomas (PCNSLs), and metastases (METs) are the most common brain malignant tumors in adult (1). Their accurate preoperative characterisation is essential as their management and prognosis differ depending on the lesion type.

Advanced functional MR imaging techniques, such as diffusion-weighted imaging (DWI), diffusion tensor imaging (DTI), perfusion-weighted imaging (PWI) and proton MR spectroscopy (^1^H-MRS) are currently the benchmark for detection and assessment of brain tumors. These techniques can help in the noninvasive differentiation of these lesions, as they provide quantitative measurements representing tumor architecture, morphology, vascularity, and metabolism (2, 3). However, differentiation of these malignancies can be challenging due to the overlapping of their imaging characteristics. There are few studies focused on differentiation between GBMs, Mets and PCNSLs (2, 3).

A meta-analysis combining articles published before December 2019, showed that DWI and DTI had a moderate diagnostic value to differentiate glioblastomas from solitary brain metastases. Additionally, large-scale prospective studies were required to explore differentiation between PCNSLs and solitary brain metastases using DWI or DTI (4). A previous meta-analysis identified a too weak correlation between the apparent diffusion coefficient (ADC) measured in DTI and cell count in lymphomas, and no evidence data for metastases concluding that ADC cannot be used as a biomarker in these entities (5).

PWI provides measurements of hemodynamic parameters of tumor microvasculature and neoangiogenesis (rCBV: relative cerebral blood volume), as well as tumor capillary permeability (PSR: percentage of signal intensity recovery) (2, 6).

^1^H-MRS allows *in vivo* detection and characterization of tumor metabolites. Changes in these metabolites often precede structural abnormalities of the tumors. By means of characteristic changes in the vascular and metabolic configurations in certains tumors, the PWI and ^1^H-MRS modalities have the potential to offer a better understanding and characterization of the vascular and metabolic profile of brain tumors. The resulting multiparametric analyzes may lead to a more precise classification of these tumors (7, 8).

Histopathological assessment is currently the gold standard for brain tumor diagnosis. However, there is growing evidence that machine learning algorithms applied on quantitative MRI data can help with non-invasive brain tumor classification (9–11).

Few studies have used machine learning techniques to differentiate common malignant brain tumors (12). They were mostly only based on dichotomized classifications of MRI data. Multiclass classifications are more useful and closer to the radiologist’s reasoning in the daily clinical practice. Machine learning decision-tree is one of the data mining methods. The decision-tree procedure is a non-parametric and nonlinear method which provides a tree-based multiclass classification to develop predictive or classification models according to variables. It classifies cases into groups or predicts values of a target variable based on values of predictor or classifier variables (13). The complex relationships between perfusion and spectroscopy MRI variables and the differences related to intra-variable correlations can be further investigated by machine learning decision tree models for multiclass classification decision making.

The aim of this study was to investigate the contribution of machine learning Classification and Regression Tree (CART) models for the multiclass classification of lymphomas, glioblastomas and metastases using perfusion and spectroscopy brain MRI multiparametric data, and then to bring out the underlying key pathophysiological processes involved in the hierarchization of the decision-making algorithms of the models.

## Materials and methods

### Patients

It was a single-center observational study conducted from January 2013 to June 2020. The study was approved by the relevant Institutional Review Board (2211250v0 23/01/2019), (granting a waiver of informed consent given the retrospective nature of study). Two hundred and one patients with histopathologically proven solitary-MET, GBM or PCNSL were consecutively recruited and evaluated. Twenty-one patients were excluded because of artifacts on perfusion data (6 patients), uninterpretable spectral data (4 patients), immunocompromised condition (2 patients), and missing data (9 patients). Thus, we included 180 patients (81 women, 99 men, mean age: 65.4 ± 15.7); 77 previously untreated immunocompetent patients with PCNSL, 45 with GBM, and 58 with solitary metastases (**Table 1**).

Table 1Patient demographics and oncotype.

**Table:** 

A. Patient demographics			
Number	Gender *n* (%)		Mean age y (± SD)
	Female	Male	
180	81 (45.0%)	99 (55.0%)	60.4 ± 7.7

**Table:** 

B. Oncotype			
Number n (%)	PCNSL	GBM	METs
	77 (42.8%)	45 (25.0%)	58 (32.2%)
Mean age y (± SD)	61.2 ± 8.0	62.1 ± 10.7	57.7 ± 7.6
Ratio M/F	1.57	1.65	0.87

PCNSLs, Primary central nervous system lymphomas; GBMs, Glioblastomas; METs, Metastases.

### Imaging protocol

All patients initially underwent a brain ^1^H-MRI examination using a whole-body system (Verio 3T; Siemens, Erlangen, Germany) with 32-channel phased-array head coil. The MRI examination was carried out strictly according to the same protocol and before the histopathological examination and/or treatment.

*Conventional MR imaging.* Protocol was the following: sagittal 3D-FLAIR (TR/TE/TI = 5000/402/1800ms, FOV= 260 mm, matrix = 156 x 128), axial 3D-T1 postcontrast (TR/TE/TI = 1900/2.93/900ms, flip angle = 9°, FOV = 255 mm, matrix = 256 x 256), and axial postcontrast T1-weighted FSE imaging (TR/TE = 308/2.48 ms, FOV = 220 mm, matrix = 272 x 352).

*PWI.* Dynamic-susceptibility perfusion contrast-enhanced T2*-weighted gradient-echo echo-planar images (TR/TE = 1980/30 ms, 4.0-mm thick sections, 0.8-mm gap, FOV = 220 mm, matrix = 128 x 128, flip angle = 90°, phases = 75) were acquired during the first pass of a standard-dose (0.1 mmol/Kg) bolus of gadoteric acid at 0.5 mmol/mL. Contrast material was injected at a rate of 6 mL/s for all patients, with a 10-second delay. From 7 to 12 sections were selected on T2 FLAIR-weighted images, depending on the volume of the tumor.

*^1^H-MR Spectroscopy.* Single voxel ^1^H-MRS was performed after intravenous administration of gadoteric acid using a point-resolved spectroscopic sequence (PRESS: TR = 1500ms/TEs = 35/135 ms, 156 scans, voxel size 15mm^3^). To avoid contamination of the voxel, saturation bands have been placed all around the voxel. Location of the voxel were determined from 3D-T1 postcontrast images on the three orientation planes, in order to reduce intravoxel dephasing by excluding brain regions such as adjacent scalp, skull, sinuses and orbits. For each kind of lesion, two MRS voxel were acquired, one voxel was localized in the hyperperfused region corresponding to the maximum value of rCBV and one another in the healthy contralateral brain parenchyma.

### Post-processing

rCBV measurements from DSC MR imaging data were performed using syngo.via software (Siemens). During the first pass of a bolus of contrast agent, T2*-weighted signal intensity decreased. The change in the relaxation rate (ΔR2*, ie, the change in the reciprocal of T2*) can be calculated from the signal intensity as follows: ΔR2*(t) ={-ln[S(t)/S_0_]}/TE, where *S(t)* is the signal intensity at time *t*, and *S_0_*, the unenhanced signal intensity. ΔR2* is proportional to the concentration of contrast agent in the tissue, and CBV is proportional to the area under the curve of ΔR2*(t), provided there is no recirculation or leakage of contrast agent. In general, these assumptions are violated, but the effects can be reduced by fitting a gamma-variate function to the measured ΔR2* curve. This function approximates the curve that would have been obtained without recirculation or leakage. CBV can then be estimated from the area under the fitted curve rather than from the original data. ROIs of standardized size (4.5-mm radius) were placed in regions of maximal CBV on CBV color overlay maps for targeting and were referenced to the symmetrically contralateral parenchyma (normal white or grey matter) for the calculation of relative maximal CBV, rCVB_max_ = CBV_lesion_/CBV_cntralateral_. Volume averaging with the blood vessels was carefully avoided, confounding factors in the CBV analysis were minimized, and the size of the ROIs was kept constant. Because the maximum perfused regions of gliomas may imply aggressiveness (2, 6, 14), we analyzed the PWI data from the maximally perfused regions of the tumors by drawing from 5 to 27 ROIs to cover the entire tumor volume, depending on the tumors.

PSR measurements were performed using syngo.via software. ROIs were drawn on the grey-scale perfusion maps overlaid on contrast-enhancing tumor on T1-weighted images. An ROI of 30–40 mm^2^ was moved within the tumor area to look for the highest and lowest recoveries on T2*-weighted signal-intensity curves and was selected for maximum and minimum PSR, respectively. For normalization, ROI of approximately 30–50 mm^2^ was also placed in the symmetrically contralateral parenchyma, and ratios were obtained. The PSR was calculated as described by Cha et al. (15): PSR = 100% x (S_1_ – S_min_)/(S_0_ - S_min_), where S_1_, S_0_, and S_min_ are post-contrast, pre-contrast and minimum T2*-weighted signal intensities, respectively.

1H-MR spectroscopy raw data were analyzed with jMRUI (Java-based Magnetic Resonance User Interface) post-processing software. Signal intensity of each metabolite was obtained using the AMARES algorithm (Advanced Method for Accurate, Robust and Efficient Spectral fitting of MRS data with use of prior knowledge) for accurate relative quantification. AMARES is a time domain curve-fitting approach, in which the zero-order phase and delay time are modeled parameters of the Lorentzian function. In this interactive quantitation method, linewidths and concentrations are part of a non-linear model and are optimized by fitting the *in vivo* signal with a combination of metabolite signals by non-linear least square techniques. The absolute concentration of metabolites from signal intensity as derived by JMRUI can be fitted to a simplified equation as published (16). To ensure short and long TE MRS methods have pros and cons *in vivo* metabolite quantification. Measurements with a long TE, under a long enough TR, may allow acquisition of the full metabolite signal and is preferable to ensure accurate quantification of metabolites such as choline (Cho), creatine (Cr), N-acetylaspartate (NAA). Due to its co-resonance with long-chain lipids (Lip), (Lip/Lac) at short TE, lactate (Lac) was quantified at long TE (lactate inverted due to the J coupling) and lipids in the following way [lip/lac]_shortTE_ - [lac]_longTE_ + [Lip]_shortTE_, thus allowing the separation and the correct quantification of lactate and lipids. Five well-resolved resonance peaks were fitted: Cho (3.22ppm), Cr (3.02ppm), NAA (2.02ppm), Lac (1.33ppm) at long TE (135ms), and Cr and Lip/Lac (1.3ppm), Lip (0.9ppm) at short TE (35ms), and their ratio were calculated Cho/NAA, Cho/Cr Lac/Cr, Lip/Cr. The data were normalized using the contralateral Cr resonance signal from the symmetric healthy parenchyma.

Spectral quality was examined for each subject based on the following parameters: fullwidth at half maximum (FWHM) as an estimation of the line width of the *in vivo* spectrum (values in ppm were converted into Hz); SNR as determined by the maximum signal and the residuals of the fitted spectrum; and peak coalescing. The subjects were excluded from the study when there were major alterations of spectral quality.

The structural image-processing tool FSL (http://www.fmrib.ox.ax.uk/fsl) was used to estimate the gray matter, white matter and CSF content of each voxel and to correct the partial volume effects on the metabolite data. Brain tissue images were extracted by removing the outer skull and scalp surfaces using the FSL Brain Extraction Tool (http://fsl.fmrib.ox.ac.uk/fsl/fslwiki/BET). Finally, the FAST/FIRST tool (http://fsl.fmrib.ox.ac.uk/fsl/fslwiki/FAST; http://fsl.fmrib.ox.ac.uk/fsl/fslwiki/FIRST) was used to calculate the segmented tissue percentage in the VOI. Coregistration between the spectroscopic VOI and the segmented image was performed with a user-developed Matlab program (MathWorks, Natick, Massachusetts).

### Decision tree model

Classification and Regression Tree (CART) models provide the combinations of the most discriminating variables in a hierarchical order according to an algorithm. Decision tree procedure consists of recursively partitioning data according to a relationship between the predictors or classifiers and response values. These partitions of the data are done recursively to form a tree of decision rules that involve the value of the input variables. The decision rules are arranged hierarchically in a tree-like structure with nodes connected by lines. The nodes represent decision rules, and the lines order the rules. The first rule at the top of the tree is called the root node, subsequent rules, internal nodes, and end rules, end (leaf) nodes with only connection (17). The partition algorithm searches all possible splits of predictors or classifiers to best predict or classify the response. It chooses optimum splits from many possible splits until the desired fit is reached.

For continuous predictors or classifiers, the partition is done according to a splitting “cut” value for the factor. The sample is divided into values below and above this cutting value. For categorical responses, decision rules provide the rate of observations, and the fitted value attributed to the response that occurred for that node of the tree for each response level. The fitted value is the probability estimated for the response levels, minimizing the residual log-likelihood chi-square. Node splitting is based on the LogWorth statistic [-log10(chi-squared p-value)] (18).

In the decision-tree, the root node is the predictor or classifier variable the most important. It divides the whole population with the highest information gain. The internal nodes are the variables classified in descending order of importance of information gain (19).

### Statistical analysis

The variables assessed were spectroscopic concentration ratios (Lac/Cr, Cho/NAA, Cho/Cr, and Lip/Cr), perfusion parameters (rCBVmax, PSRmax), and oncotype groups (METs, GBMs, PCNSLs). Mean values and frequencies were expressed with their standard deviations (± SDs) and percentages (%), respectively. Kolmogorov–Smirnov test confirmed normal distribution of continuous variables.

Differences among groups were tested using 1-way ANOVA and the Fisher PLSD *post hoc* test (Fisher’s Protected Least Significant Difference).

Data mining algorithms, especially decision-tree, works with no missing data. Therefore, after cleaning and preparing the dataset, only 180 patients were included in the final data analysis.

All the variables were included at the same time as input for the Classification and Regression Tree models. The K fold random cross-validation procedure to train and test classifiers was used with K = 5 to improve the estimated performance of the model, although it is computationally expensive to train the model on multiple training sets. There is a bias-variance trade-off associated with the choice of k in k-fold cross-validation. Typically using k = 5 yields test error rate estimations that suffer neither from excessively high bias error nor from very high variance error. K-fold cross validation randomly splits the dataset into k stratified folds. Iteratively, each of the k sets is used as a test set once from new unseen data to validate the model while the remaining data (k-1 folds) are used as a training set to fit and generate the model. The process is repeated K times, i.e., as many times as the number of stratified folds. Thus, each data point is used in a test set only once. In total, k models are generated, and k validation statistics are obtained. To prevent overfitting which is the risk of good model validation on training data and poor model generalization on testing data, the k-fold cross-validation stopping rule is a minimum threshold for improvement in the cross validation RSquare. The stopping rule selects a model for which the next ten models have a cross validation RSquare improvement of less than 0.005 units. The model giving the best tradeoff between bias error and variance error is chosen as the final model. This method is useful for small data sets, because it makes efficient use of limited amounts of data.

In our study, the Classification and Regression Tree (CART) models are the decision-tree algorithms which were applied for brain tumor multiclass classification. They create a division of the tree and pruning a tree on the cost complexity (20). Information Gain, Gini index and Gain ratio are important splitting criteria. The CART algorithm uses the Gini impurity index to select the best variable.

Impurity was measured by the Gini index as:


$$Gini\;\left(D\right)=1-\sum_{i=1}^{m}P_{i}^{2}$$

with $P_{i}$ is the probability recording in *D* belongs to class *Ci* and is estimated by $\frac{\left|Ci,D\right|}{\left|D\right|}$ (20). The sum is computed over *m* classes.

A confusion matrix was constructed to determine the performance of the decision-tree process in the multiclass classification procedure of oncotype group variable, and the corresponding accuracy, sensitivity, specificity, positive predictive value (PPV), and negative predictive value (NPV) were calculated. In addition, the RSquare value, the accuracy and the classification performance quantified by the area under the receiver operating characteristics (ROC) curve (AUC) with 95% confidence interval (CI), were computed for comparison (21).

The ROC plot is a method to visualize and select classifiers based on their classification performance as quantified by the area under the ROC curve (AUC). The classifier’s area under the curve (AUC) can be described as the classifier’s probability of classifying a randomly selected positive result with the highest predictive accuracy.

Statistics were performed using SAS software (version 9.4; SAS Institute, Carry, NC). A two-tailed *P*-value< 0.05 was considered statistically significant.

## Results

### Perfusion and Spectoscopy parameter analysis

**Figure 1:**
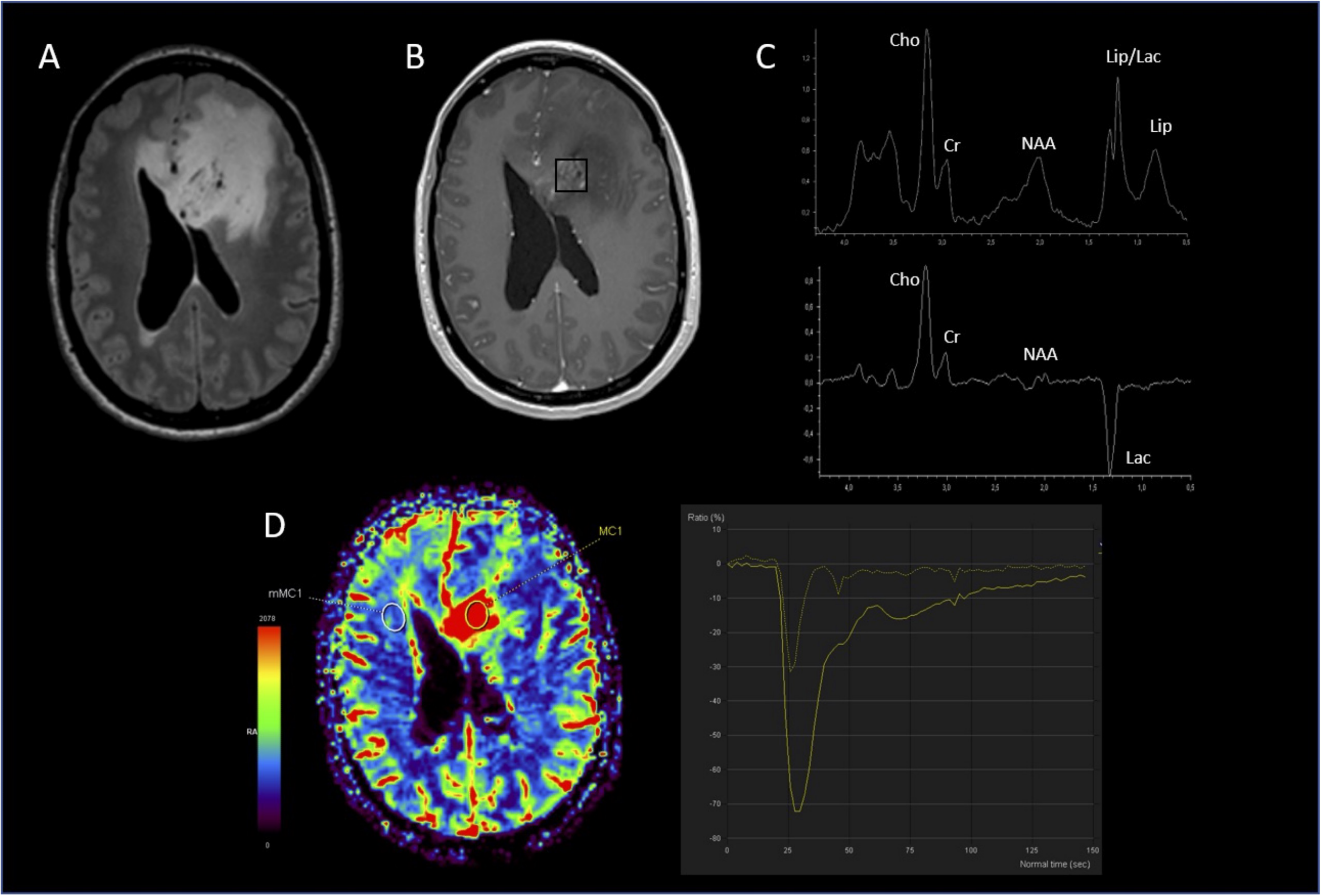
Glioblastoma. **(A)** hypersignal FLAIR of the lesion; **(B)** location of the MRS voxel in the enhancing signal on the 3D T1 post-gadolinium; **(C)** spectrum of the MRS voxel obtained at short TE (top) and long TE (bottom) showing a strong resonance of lactate and increased Cho/NAA & Cho/Cr ratios; **(D)** PWI CBV cartography showing hyperperfusion of the lesion with a rCBVmax at 4.56.

**Figure 2:**
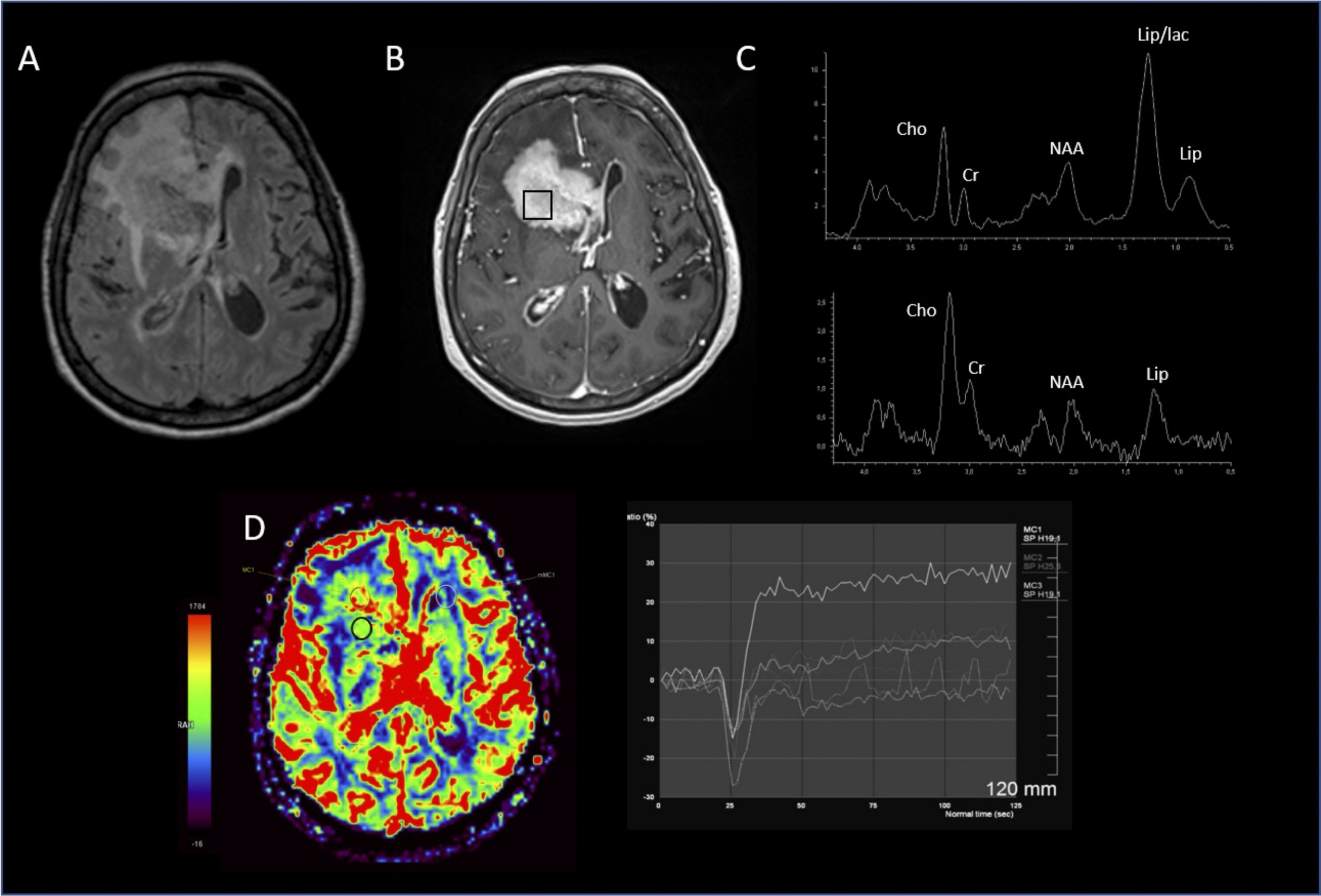
Lymphoma. **(A)** hypersignal FLAIR of the lesion; **(B)** location of the MRS voxel in the enhancing signal on the 3D T1 post-gadolinium; **(C)** spectrum of the MRS voxel obtained at short TE (top) showing a strong resonance of Lipid and long TE (bottom), increased Cho/NAA & Cho/Cr ratios; **(D)** PWI imaging showing PSR about 180%, without significant increasing rCBV.

**Figure 3:**
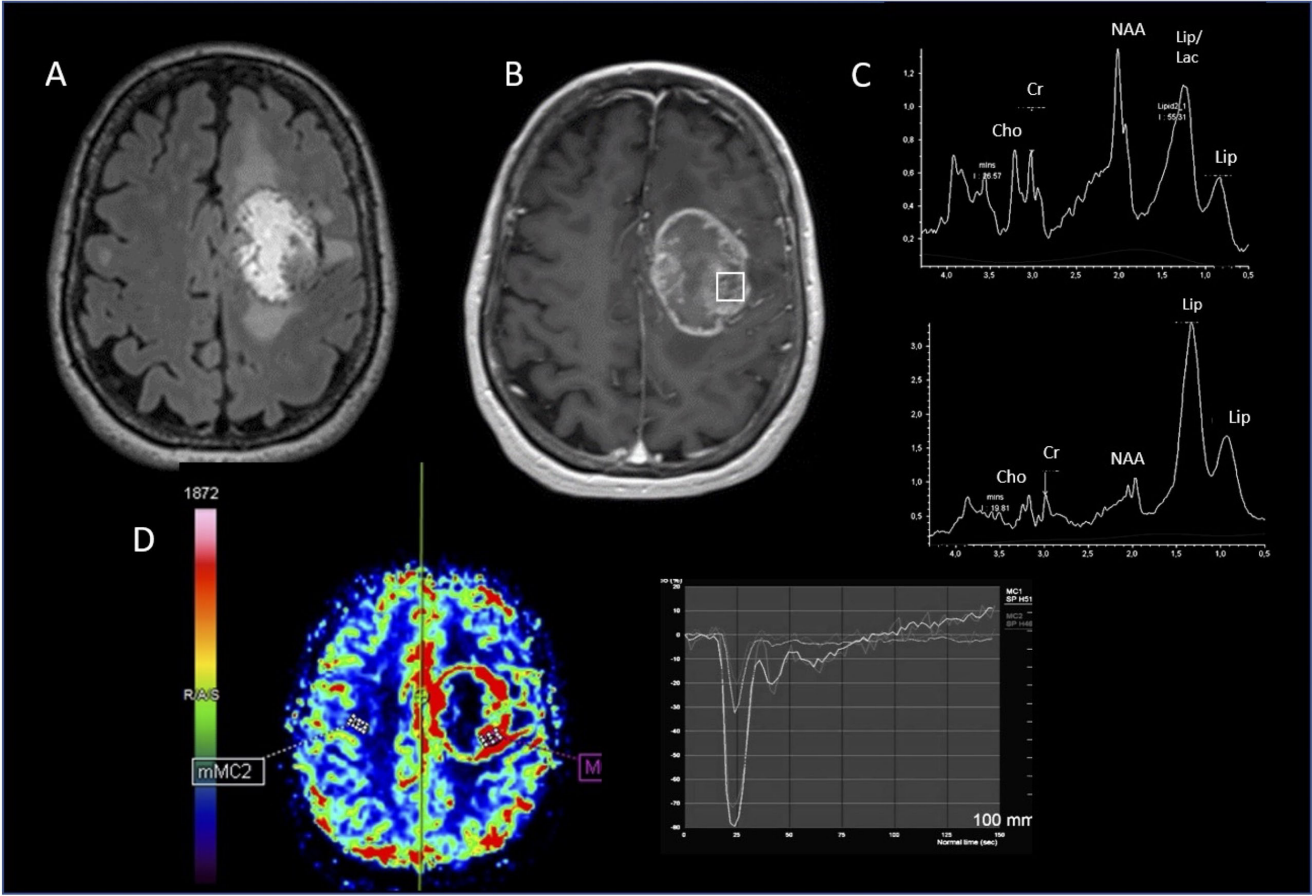
Metastasis. MRI-MRS imaging. **(A)** hypersignal FLAIR of the lesion; **(B)** location of the MRS voxel in the enhancing signal on the 3D T1 post-gadolinium; **(C)** spectrum of the MRS voxel obtained at short TE (top) showing a strong resonance of Lipid and long TE (bottom) increased Cho/NAA & Cho/Cr ratios persistence of free lipids resonance; **(D)** PWI cartography of CBV showing hyperperfusion of the lesion with a rCBVmax at 2.96.

Mean values of perfusion parameters (rCBV, PSRmax) and spectroscopic concentration ratios (Cho/Cr, Cho/NAA, Lac/Cr, Lip/Cr) in the tumor groups PCNSL, GBM, and metastases are depicted in **Table 2**. One-way ANOVA of variance analysis applied to the perfusion and spectroscopy parameters showed significant differences among the 3 groups of lesions, for all studied parameters (P<.001, respectively). Pairwise comparisons for the one-way ANOVA using Fisher’s PSLD test are summarized in **Table 2**. Perfusion and spectroscopy MRI images for GBMs, PCNSLs and METs are displayed in **Figures 1**–**3**, respectively.

**Table 2:** Mean values and standard deviation (SD) of perfusion and spectroscopy parameters in the differentiation of brain tumours.

Oncotype of lesions	rCBV	PSRmax	Cho/Cr	Cho/Naa	Lact/Cr	Lip/Cr
PCNSLs	1.8536 ± 0.503	107.145 ± 13.305	3.283 ± 1.3449	2.415 ± 1.345	2.469 ± 1.333	3.039 ± 1.582
GBMs	2.470 ± 0.998	85.522 ±12.456	2.932 ± 1.337	3.157 ± 1.337	1.796 ± 0.929	4.167 ± 2.245
METs	1.655 ± 1.057	86.647 ± 26.853	2.196 ± 1.342	-*	0.807 ± 0.917	6.193 ± 2.004
*P* (anova)	< 0.001	< 0.001	<0.001	<0.001	<0.001	<0.001
F (anova)	18.216	27.908	10.985	110.194	36.691	45.659
*P* PCNSLs vs GBM	*< 0.001*	*< 0.001*	0.149	*0.006*	*<0.001*	*0.001*
*P* PCNSLs vs METs	0.191	*< 0.001*	*<0.001*	–	*<0.001*	*<0.001*
*P* GBM vs METs	*< 0.001*	0.811	*0.013*	–	*<0.001*	*<0.001*

*No measurable NAA peaks at 2.02 ppm in METsastases.

### Classification and Regression decision-Tree (CART) algorithm for multiclass classification of PCNSLs, GBMs and METs

**Figure 4:**
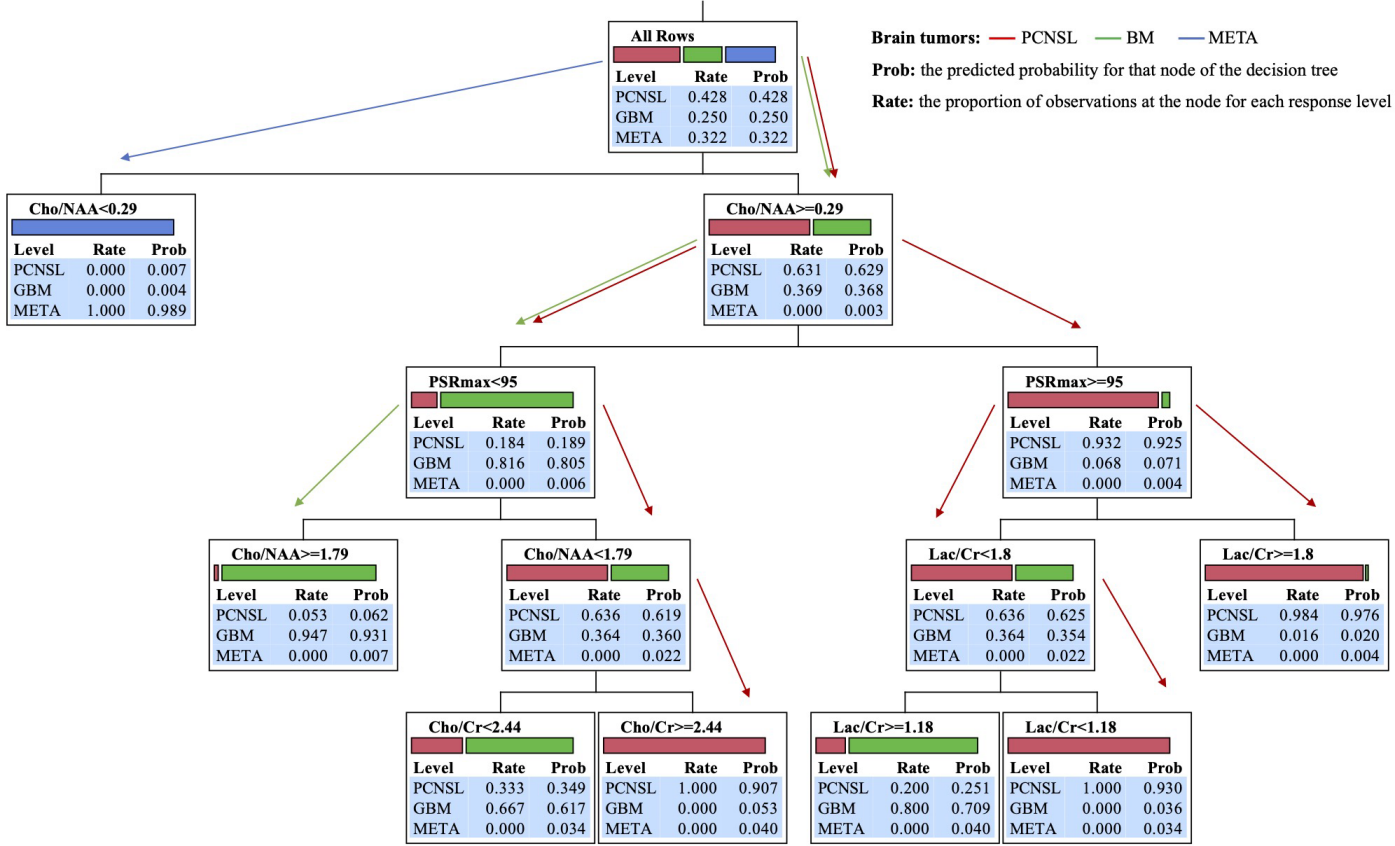
Block diagrams of analysis steps in machine learning decision-tree models for multiclass classification of PCNSL, GBM, and MET brain tumors.

**Figure 5:**
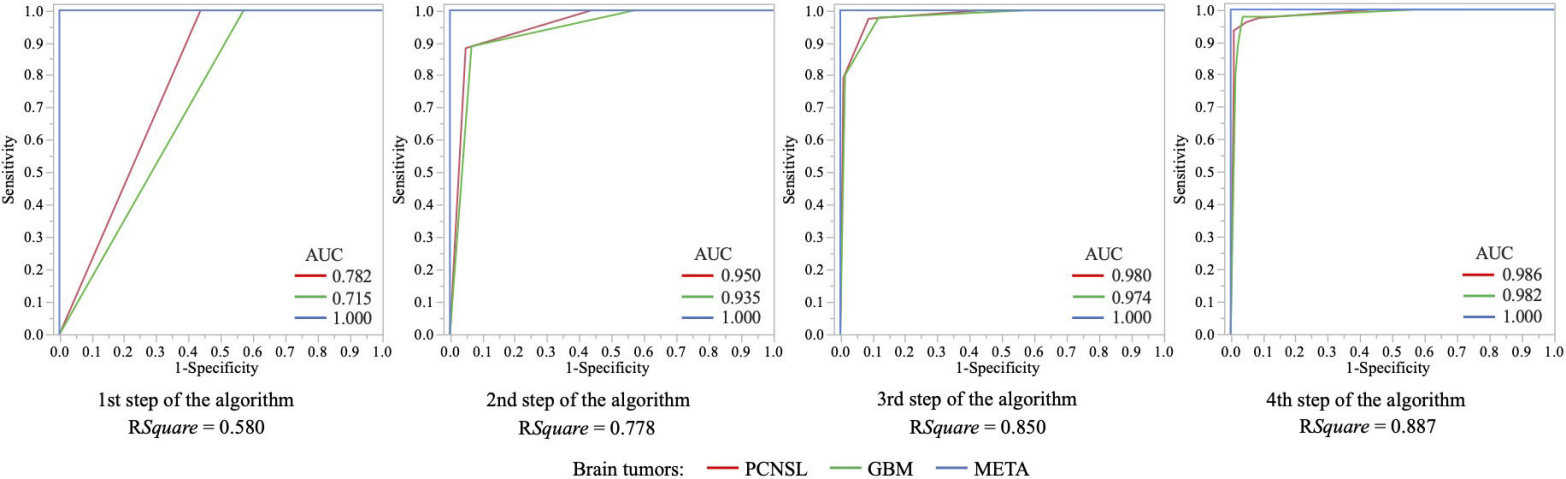
ROC graphs visualizing and selecting classifiers based on their classification performance quantified by the areas under the ROC curve (AUC) for each step of the algorithm.

The CART decision-tree model successfully classified the 3 tumor types in our cohort (**Figure 4**). The performance (AUC) of the models was 0.98 for PCNSLs, 0.98 for GBM and 1.00 for METs (**Figure 5**). The accuracy of the model was 0.96 with a RSquare of 0.887.

In hierarchical order of importance, the nodes in the decision-tree model algorithm were Cho/NAA, PSRmax, and Lac/Cr or Cho/Cr for the multiclass classification decision making of PCNSLs, GBMs, and METs brain tumors. The block diagram in **Figure 1** summarizes the analysis steps in decision-tree and machine learning models for multiclass classification of all tumors. Five rules of classifier combinations were extracted through the decision tree model:

Rule 1 was Cho/NAA< 0.29 with a predicted probability of 0.989 for that node of the tree for META multiclass classification.

Rule 2 was Cho/NAA ≥ 1.79, PSR< 95 with a predicted probability of 0.931 for that end node of the tree for GBM multiclass classification.

Rule 3 was Cho/NAA [0.29 - 1.79[, PSR< 95, Cho/Cr ≥ 2.44 with a predicted probability of 0.907 for that end node of the tree for PCNSL multiclass classification.

Rule 4 was Cho/NAA ≥ 0.29, PSR ≥ 95, Lac/Cr< 1.18 with a predicted probability of 0.930 for that end node of the tree for PCNSL multiclass classification.

Rule 5 was Cho/NAA ≥ 0.29, PSR ≥ 95, Lac/Cr ≥ 1.80 with a predicted probability of 0.976 for that end node of the tree for PCNSL multiclass classification.

## Discussion

We investigated the contribution of Classification and Regression Tree (CART) models in the multiclass classification of lymphomas, glioblastomas, and metastases where metabolic and perfusion data are used as classification features.

### Decision-tree models

Decision-tree models are modeling methods that have several advantages. They can handle nonlinear complexe relationships, creating rules, and being easy to interpret (22, 23). They have the ability to convert complex risk equations into an organized flowchart, which can be easily navigated to identify appropriate classifiers. A simple, practical, and user-friendly approach which can help clinicians to make more valid classifier-based decisions. Furthermore, they help to remove unnecessary parameters to classify brain tumors.

The strengths of decision tree models are the prioritization in order of importance of classifiers in multiclass classification decision making and the visualization of the risk or class of a given subject. This can help in better decision making than predictors obtained from regression models (24). This is a clear advantage of decision-tree models compared to multivariate regression models. Indeed, decision-tree models reveal the classifiers that have the highest classification accuracy, and yield threshold values of these classifier where tree branching takes place. One key feature is that the developed decision-tree models in this study provide a hierarchical organization of the different classifiers in brain tumor multiclass classification. The most sensitive classifiers appear first in the decision tree and the most specific classifiers last. This allows a hierarchical utilization of the different classifiers for multiclass classification decision making. Moreover, the decision-tree model yields a supervised machine learning classification that can accurately discriminate classifiers based on training dataset, retrospectively acquired, and can assesse the generalization of classifiers on a testing prospective dataset.

Nonlinear classification of data, which involves multiple classes in the real world, is a crucial research topic in the field of data classification (25–28). Deep networks have proven highly efficient in executing numerous complex tasks, such as nonlinear multiclass classification of data and images, but at the expense of interpretability (29–32). Besides, tree-based models are widely used at predictive or classification tasks using structured tabular data (28, 33). Lack of interpretability limits the use of deep networks in applications involving clinical decision making. Transparency of the decision-making processes of deep networks remains an essential clinical, legal, and ethical requirement for patient care (34). In this context, some recent research explores novel alternative approaches to decision tree training that aim to improve the performance, interpretability and ease of implementation of models in nonlinear data multiclass classification (28, 33, 35, 36). In these avenues for future work, decision tree-based deep neural networks aims to combine the concepts of deep networks and decision tree models to facilitate more accurate and robust nonlinear classification of data (28). This also aims to provide a better understanding of deep networks and paves the way for the transparency of their black box nature (35). Prospects for expanding the application of deep networks for multiclass classification of common malignant brain tumors using MRI data holds immense potential for various advancements in the field.

### Algorithm nodes of decision-tree models in hierarchical order of importance

In hierarchical order of importance, algorithm nodes of our Classification and Regression Tree (CART) models were Cho/NAA, PSRmax, and Lac/Cr or Cho/Cr for decision making of PCNSLs, GBMs, and METs tumor multiclass classification.

### The root node of the algorithm

The root node (Cho/NAA) of the algorithm was based on the underlying metabolic tumor process involved in membrane lipid metabolism, due to increased cell membrane turnover by proliferating tumor cells, versus neuronal density and viability. The root node led to the decision making of the MET classification.

Indeed, Cho is phosphorylated by Choline Kinase (CK) to phosphocholine (P-Cho) through the CDP-choline pathway of phosphatidylcholine (PtdCho) biosynthesis (37, 38). P-Cho for PtdCho synthesis can also be produced from sphingomyelin hydrolysis by a lysosomal sphingomyelinase (39). PtdCho is considered as an intermediate in the cycle of synthesis and degradation of subcellular membrane lipid. The enzyme Choline Kinase is overexpressed in several brain tumors, hence the presence of choline peak in MRS spectra, which reflects an increased cell membrane turnover and proliferation, as well as increased cellularity (38). In tumors, the level of Cho correlates with the malignancy degree, reflecting cellularity (40). However, some studies have reported that the Cho/Cr ratio alone is not reliable in differentiating brain METs from GBMs (1).

NAA peak gets contributions from N-acetylaspartyl glutamate (NAAG), glycoproteins, and amino acid residues in peptides (37). NAA is one of the most abundant amino acids in the CNS (41, 42). It is synthesized in the neuronal mitochondria by the L-aspartate *N*-acetyltransferase from L-aspartate and acetyl coenzyme A, and is transported into the cytosol to be converted by the enzyme aspartoacylase into aspartate and acetate (41, 42). NAA is a marker of neural density and functional integrity of neuronal mitochondrial metabolism (42). NAA decrease indicates a loss of neuronal structures or function, or a displacement of normal brain tissue by highly proliferating tumor cells (37, 43). NAA is not present in tumors outside the central nervous system (44).

The close relationship between Cho and NAA may explain the importance of the assessment of membrane lipid metabolism versus neuronal viability (Cho/NAA ratio) for the classification decision making according to the infiltration or displacement of normal brain tissue relative to neuronal density.

In this study, significantly elevated Cho/Cr levels were noted in PCNSLs compared to those in METs (P< 0.001). The Cho/Cr ratio in GBMs was also significantly higher than that in METs (P = 0.013), but was not significantly different than that in PCNSL (P = 0.0149), in agreement with several previous studies (1–3). NAA was undetectable at 2.02 ppm in METs. Significantly elevated Cho/NAA levels in GBMs compared with those in PCNSLs (P = 0.006) indicated that loss of neuronal structures or functions was higher in GBMs than that in PCNLs.

Indeed, NAA levels are almost completely absent in brain metastases (45, 46). They constitute a strong argument in the multiclass classification of tumors and may partly explain the good performance of the model. Typically, metastases grow expansively and noninfiltratively, and rarely contain brain tissue within the lesion. Metastatic lesions tend to be encapsulated without high Cho signals or other abnormalities outside the region of enhancement (8, 47). The brain is replaced by the lesion (48) and the presence of NAA in the spectra of metastases is attributed to voxel contamination by adjacent normal brain rather than to the intra-tumor signal (44, 48–50). On the other hand, GBM and PCNSL tumors are not circumscribed but diffusely infiltrating into the brain tissue. NAA is decreased whenever the brain is damaged. The infiltrated brain parenchyma can produce NAA signals without definite contamination from surrounding normal brain tissue (49).

The root node of the algorithm was primarily based on the proliferative, infiltrative, and neuronal destructive characteristics of the tumor.

### The second node of the algorithm

The second node of the algorithm (PSR_max_) concerned the underlying hemodynamic tumor process involved in capillary permeability.

PSRmax is the percentage of maximum signal intensity recovered at the end of the first pass of contrast agent relative to baseline (before contrast administration) from the T2* signal-intensity curve of MRI perfusion (2). The degree of this recovery depends on several factors, such as contrast agent leakage, extravascular space size, and blood flow rate (2).

In this study, significantly elevated PSRmax levels were noted in PCNSLs compared to those in GBMs and METs (P< 0.001, respectively), without significant difference between those in GBMs and METs (P = 0.811). Previous studies (2, 14, 15, 51) showed that tumor capillary permeability assessment (PSRmax) appears to be useful in differentiating PCNSLs from GBMs and METs, or METs from PCNSLs and GBMs. Furthermore, combined Cho/NAA and PSRmax classifier may provide the best differential diagnostic performance to discriminate PCNSLs from GBMs and METs (3). However, PSRmax on its own is not effective enough to discriminate GBMs from other common brain tumors such as PCNSLs and METs (2, 3, 14, 15, 51).

The second node of the algorithm was primarily based on tumor tissue capillary permeability characteristics.

### The end node of the algorithm

The third and end node of the algorithm hinged on the underlying metabolic tumor process involved in intracellular glycolytic energy metabolism (Lac/Cr) in the decision making of GBMs and PCNSLs classifications, or membrane lipid metabolism due to increased cell membrane turnover by proliferating tumor cells (Cho/Cr) for PCNSLs classification decision making.

Lactate (Lac) is usually undetectable by MR imaging in a healthy brain. Its detection indicates the presence of ischemic processes and macrophage invasion (52). Lactate detection indicates an alterated cell energy metabolism related to increased energy demand (52). Lactate resonance (Lac/Cr) indicates impaired oxidative phosphorylation and increased anaeroby glycolysis (Warburg effect) (53, 54) linked to a highly cellular process, associated to increased membrane lipid metabolism (Cho/Cr) due to membrane biosynthesis by proliferating tumor cells (52). In brain tumors, lactate is an indicator of malignancy (52, 55, 56).

In the present study, significantly elevated Lac/Cr levels were noted in PCNSLs compared to those in GBMs (P< 0.001), themselves greater than those in METs (P< 0.001), which is consistent with several studies (51, 52). A previous study showed that combined PSRmax and lactate/Cr or PSRmax and Cho/Cr classifiers may provide the best differential diagnostic performance to discriminate METs from PCNSLs and GBMs (3). However, Lactate resonance on its own has not been found reliable in distinguishing the brain METs from GBMs (47, 57, 58).

The end node of the algorithm was primarily based on energy glycolytic (Warburg effect) or membrane lipid tumor metabolism.

Cerebral blood volume (rCBV) is an index of microvascularity and neoangiogenesis correlated with the aggressiveness and malignancy of tumors (2, 6). It has been used in the grading of gliomas (14).

Significantly elevated rCBV levels were noted in GBMs compared to those in METs and PCNSLs, (P< 0.001, respectively), which is consistent with several studies (2, 14, 15, 59, 60). However, there is a substantial overlap in rCBV values between these malignancies. Several prior studies have reported that rCBV have highlighted some salient features of these malignancies, but they are not always consistent and corroborative. Accordingly, rCBV may not be useful in the discrimination between these tumor types (2, 14, 15, 51, 59, 61, 62). In the case of metastases, rCBV values may vary over a large range depending on the origin and histopathology of the tumor tissue. Hypervascular metastases such as renal cell carcinoma and melanoma have a markedly high rCBV than less vascularized metastases (60, 63). PSR has been reported to be a better criterion than rCBV to differentiate these lesions (2, 3, 14, 51, 64).

As a result, microvasculature and neoangiogenesis tumor (rCBV) was not a sufficiently efficient algorithm node for multiclass classification of common brain tumors.

### The strengths and limitations of the study

Our study has several potential strenghs including histopathologic examination to confirm the diagnosis in all patients prior to inclusion, a multiclass classification procedure (three-class classification), K-fold random cross-validation to minimize the risk of overfitting (high variance error and low bias error) and provide a better approximation of the performance of the trained model in clinical practice. The interesting property of a machine learning model is its ability to categorize new unseen data. Cross validation is a technique used to determine how the results of a machine learning model could be generalized to new, unseen data. This approach provides a mechanism to get the test-data Mean Square Error with the current dataset without the need of finding new data to test the model.

The implementation of machine learning decision tree models focused on data from the relevant MRI modalities, such as perfusion and spectroscopy, for the multiclass classification of lymphomas, glioblastomas and metastases. This was to highlight and understand the involvement of the relevant underlying hemodynamic and metabolic pathophysiological processes in the prioritization of machine learning decision tree model discrimination algorithms for the multiclass classification of these brain tumors. The information contained in the multiparametric data of perfusion and spectroscopy constituted a sufficiently enriched informative value to allow a satisfactory multiclass classification performance of these brain tumors. This could suggest that there was little to be gained by introducing additional data from less relevant modalities.

Our study may have a few potential limitations including small sample size, model generalizability from model performance monocentric validation, quantification of the metabolites’ concentration which may be affected by low signal-to-noise ratio, field inhomogeneities, metabolite spectra overlapping, and quantification of rCBV in regions of disrupted BBB with consecutive leakage of contrast agent into the interstitial space which may affect its accuracy (14, 51). In regions of disrupted BBB with consecutive leakage of contrast agent into the interstitial space, rCBV measurement accuracy may be affected. The increase in the T1 weighted signal from contrast media leakage can partly cancel out the decrease in the T2* weighted signal, which can lead to an over- or under-estimation of perfusion parameters (65). There are several strategies to minimize the effect of T1 shortening, such as preload contrast agent administration or mathematic leakage-correction model (66). However, the lack of consensus reflects that methodical flaws are not yet resolved limiting the comparability of different MR perfusion studies.

## Conclusion

Our study shows potential implementation of machine learning algorithms and decision tree-models based on perfusion and spectroscopy MRI data for an accurate multiclass classification of common brain tumors. The complex and nonlinear relationships between many perfusion and spectroscopy MRI variables can be simplified by multivariate classification methods, and differences related to intra-variable correlations may be further emphasied between tumor types. Classification and Regression Tree models allow a hierarchical and convenient use of MRI perfusion and spectroscopy parameters for the multiclass classification of these brain tumors and provide a supplementary approach for a personalized decision support.

## Data availability statement

The raw data supporting the conclusions of this article will be made available by the authors, without undue reservation.

## Ethics statement

The studies involving human participants were reviewed and approved by Swiss National Research Committee. The patients/participants provided their written informed consent to participate in this study.

## Author contributions

RV: conception and design of the study, data analysis, writing of the manuscript. J-NV: conception and review of the manuscript. CG: data collection and review of the manuscript. AL: review of the manuscript. CT: data collection and review of the manuscript. GR: review of the manuscript. MW: review of the manuscript. RG: conception the study, review of the manuscript, and principal investigator for the study. AV: conception and design of the study, and review of the manuscript. All authors contributed to the article and approved the submitted version.
